# Around the Hospitals

**Published:** 1892-05-21

**Authors:** 


### Around the Hospitals.

**The German Hospital, Dalston,** which ministers to over 1,500 in, and 24,757 out-patients during the year, is in a better way, financially, than most of our hospitals, as shown by the balance-sheet in the annual report just issued. The institution has many warm friends, amongst them being the German Emperor, who, on the occasion of the festival dinner lately held, contributed £200 of the £2,862 collected on this occasion.

**The British Home for Incurables** in the Clapham Road, is one of the most useful of its kind in the Kingdom. It not only provides a home for those eligible for admission, but affords pensions to sufferers from epilepsy and cancer. These annuities amount to £20 each, and 550 patients have been elected to receive them since the foundation of the home in 1861. The immense boon such a form of charity is, where a hopeless invalid is thrown on the limited resources of a poor home, cannot be over estimated. The expenditure on the in-patients is necessarily large, amounting to £100 per annum per patient, and as the home does not share in the Hospital Saturday Fund, private subscriptions are much required. The institution is contemplating new buildings, and for these a site has already been secured at Streatham. At the annual dinner lately held at which Earl Amherst presided, the large sum of £4,691 was announced as collected on the occasion.

**The Great Northern Central Hospital** has passed through a year of great activity, and during that period admitted 1,072 in-patients, and 17,350 out-patients, the largest number of admissions since the establishment of the institution. It is satisfactory to note that the expenditure has not increased correspondingly. Side by side with this prosperity we have to regret the loss of many kind friends of the hospital. The late Duke of Clarence, president of the institution, was to have laid the foundation stone of new buildings this year. These additions are so urgently needed that the Committee have decided to commence the work immediately, although the whole sum needed for the purpose has not yet been subscribed, and £14,000 are still required. The want of a convalescent home in connection with the hospital is much felt, and the Committee are dependent on the kindness of several other institutions for the reception of patients after treatment in the hospital. The Ladies' Association is of immense assistance in attending to the welfare of patients in their own homes. Such associations would, with advantage, supplement all institutions for the relief of the sick poor.

**The London Fever Hospital** report is pleasant reading in many ways. It amply proves that the more dangerous fevers have, to a considerable extent, been robbed of the dire consequences that used almost invariably to follow an attack. It is really marvellous that the mortality among the 339 patients treated for scarlet fever in the Hospital last year should have been kept down to the extremely low average of 0·9 per cent.; and this fact reflects the greatest credit upon both the medical staff and nurses. All the more gratifying is this mortality when compared with the average mortality from the same disease—5·1 per cent.—for all London. What wonder then that the benefits of this Institution are more and more appreciated by those who can afford to pay a small part of the expenses of their treatment. Another fact for which the hospital managers deserve a word of praise is that not one patient contracted any other disease while in the Institution. This shows the great care exercised to see that each patient is taken in the proper ward, and is a testimony to the professional skill of Dr. Hopwood, upon whom this duty devolves. We are largely indebted to the London Fever Hospital, and we must not forget that London may at any time be visited with a fever epidemic, so that it is of paramount importance that a hospital, which has shown such wonderful satisfactory results, should be supplied with ample funds to keep its organisation in thorough working order, and thus be ever ready to deal with any emergency. For these reasons we trust this most necessary and useful institution may be more liberally supported.

